# Differential immunophenotype of circulating monocytes from pregnant women in response to viral ligands

**DOI:** 10.1186/s12884-023-05562-0

**Published:** 2023-05-06

**Authors:** Marcelo Farias-Jofre, Roberto Romero, Yi Xu, Dustyn Levenson, Li Tao, Tomi Kanninen, Jose Galaz, Marcia Arenas-Hernandez, Zhenjie Liu, Derek Miller, Gaurav Bhatti, Megan Seyerle, Adi L. Tarca, Nardhy Gomez-Lopez

**Affiliations:** 1 Pregnancy Research Branch, Division of Obstetrics and Maternal-Fetal Medicine, Division of Intramural Research, Eunice Kennedy Shriver National Institute of Child Health and Human Development, National Institutes of Health, U.S. Department of Health and Human Services (NICHD/NIH/DHHS), Detroit, MI 48201 USA; 2 Department of Obstetrics and Gynecology, Wayne State University School of Medicine, Detroit, Michigan, 48201 USA; 3 Division of Obstetrics and Gynecology, Faculty of Medicine, Pontificia Universidad Católica de Chile, 8330024 Santiago, Chile; 4 Department of Obstetrics and Gynecology, University of Michigan, Ann Arbor, MI 48109 USA; 5 Department of Epidemiology and Biostatistics, Michigan State University, East Lansing, MI 48824 USA; 6 Wayne State University School of Medicine, Detroit, MI 48201 USA; 7 Department of Computer Science, Wayne State University College of Engineering, Detroit, MI 48202 USA; 8 Center for Molecular Medicine and Genetics, Wayne State University, Detroit, MI 48201 USA; 9 Department of Biochemistry, Microbiology, and Immunology, Wayne State University School of Medicine, Detroit, MI 48201 USA

**Keywords:** Virus, Innate immunity, Infection, Pregnancy, Human

## Abstract

**Background:**

Viral infections during pregnancy can have deleterious effects on mothers and their offspring. Monocytes participate in the maternal host defense against invading viruses; however, whether pregnancy alters monocyte responses is still under investigation. Herein, we undertook a comprehensive in vitro study of peripheral monocytes to characterize the differences in phenotype and interferon release driven by viral ligands between pregnant and non-pregnant women.

**Methods:**

Peripheral blood was collected from third-trimester pregnant (*n* = 20) or non-pregnant (*n* = 20, controls) women. Peripheral blood mononuclear cells were isolated and exposed to R848 (TLR7/TLR8 agonist), Gardiquimod (TLR7 agonist), Poly(I:C) (HMW) VacciGrade™ (TLR3 agonist), Poly(I:C) (HMW) LyoVec™ (RIG-I/MDA-5 agonist), or ODN2216 (TLR9 agonist) for 24 h. Cells and supernatants were collected for monocyte phenotyping and immunoassays to detect specific interferons, respectively.

**Results:**

The proportions of classical (CD14^hi^CD16^−^), intermediate (CD14^hi^CD16^+^), non-classical (CD14^lo^CD16^+^), and CD14^lo^CD16^−^ monocytes were differentially affected between pregnant and non-pregnant women in response to TLR3 stimulation. The proportions of pregnancy-derived monocytes expressing adhesion molecules (Basigin and PSGL-1) or the chemokine receptors CCR5 and CCR2 were diminished in response to TLR7/TLR8 stimulation, while the proportions of CCR5^−^ monocytes were increased. Such differences were found to be primarily driven by TLR8 signaling, rather than TLR7. Moreover, the proportions of monocytes expressing the chemokine receptor CXCR1 were increased during pregnancy in response to poly(I:C) stimulation through TLR3, but not RIG-I/MDA-5. By contrast, pregnancy-specific changes in the monocyte response to TLR9 stimulation were not observed. Notably, the soluble interferon response to viral stimulation by mononuclear cells was not diminished in pregnancy.

**Conclusions:**

Our data provide insight into the differential responsiveness of pregnancy-derived monocytes to ssRNA and dsRNA, mainly driven by TLR8 and membrane-bound TLR3, which may help to explain the increased susceptibility of pregnant women to adverse outcomes resulting from viral infection as observed during recent and historic pandemics.

**Supplementary Information:**

The online version contains supplementary material available at 10.1186/s12884-023-05562-0.

## Background

Infection during pregnancy is one of the leading causes of maternal mortality and morbidity worldwide, accounting for more than 10% of all deaths [1]. Notably, both viral and bacterial infections have been linked to adverse pregnancy outcomes [2]. Indeed, viral infection during pregnancy has been associated with increased risk of pregnancy complications such as miscarriage, stillbirth, preterm birth, pre-eclampsia, fetal growth restriction, and congenital defects, among others [3–6]. Considering past and recent viral pandemics, as well as the growing knowledge of viral infection during pregnancy, it has become evident that specific viral infections can have devastating short- and long-term effects on both the mother and offspring [7–17]. Thus, it is imperative to elucidate the underlying mechanisms whereby viral infection disproportionately impacts pregnant women to design novel preventative and therapeutic approaches.

Viruses are broadly classified by the type of carried genetic material (RNA or DNA) and display infection strategies that vary accordingly [18]. Moreover, each type of virus, together with its mechanisms of replication, requires tailored mechanisms of detection and clearance by host cells [19–22]. Importantly, although viral proteins typically elicit an intense initial immune response, the higher viral mutation rates make continuous surveillance by the host immune system challenging [23, 24]. Thus, the ability to detect general patterns of viral genetic material is a critical component of the early antiviral immune response that is primarily accomplished by innate immune cells such as monocytes [21, 25–27].

Monocytes are part of the first line of defense against pathogens, including viral infection [28–32]. These innate immune cells are equipped to detect and kill microbes, being the primary subset of circulating mononuclear phagocytic cells, and are capable of quickly secreting pro-inflammatory cytokines in response to viral encounter [33–35]. Monocytes express multiple pattern recognition receptors (PRRs), such as Toll-like receptors (TLRs), which can recognize conserved viral motifs known as pattern-associated molecular patterns (PAMPs) [27, 36]. Intracellular PRRs include TLR3, TLR7, TLR8, and TLR9, all of which are located within the endosomal membrane [37–40] and are specific for double-stranded (ds)RNA (TLR3) [41], single-stranded (ss)RNA (TLR7, TLR8) [42, 43], or dsDNA (TLR9) [44–46]. Cells also express specific PRRs within the cytosolic space, such as Retinoic Acid-Inducible Gene I (RIG-I) and Melanoma Differentiation-Associated Protein 5 (MDA5) [47], both of which detect dsRNA [48]. Interestingly, some viruses can be recognized by multiple PRRs due to their replication cycle, which includes phases wherein the virus contains both dsRNA and ssRNA [47, 49–52]. Thus, the host response to viruses is complex and requires the expression of multiple PRRs by sentinel cells such as monocytes. Given that monocytes are increased in number [53–56] and display activated phenotypes during pregnancy [56–60], such innate immune cells are likely primed to participate in maternal response to viral infection. However, the evaluation of circulating monocyte responses to different types of virus during pregnancy has not been undertaken.

Herein, we performed a comprehensive in vitro study of peripheral monocyte responses to viral genetic material mimetics in pregnant and non-pregnant women. We investigated the population distribution and expression of surface proteins (i.e., adhesion molecules and chemokine receptors) by conventional monocyte subsets (classical, intermediate, non-classical, and CD14^lo^CD16^−^) using flow cytometry. In addition, we profiled specific type I, II, and III interferons released by monocytes in response to viral ligand stimulation. Together, these data provide an overview of changes in the monocyte response to viral infection during pregnancy.

## Methods

### Human subjects, clinical specimens, and definitions

Peripheral blood samples were obtained from August 2020 – February 2021 from healthy pregnant and non-pregnant women recruited by the Pregnancy Research Branch, an intramural program of the *Eunice Kennedy Shriver* National Institute of Child Health and Human Development (NICHD), National Institutes of Health (NIH), U.S. Department of Health and Human Services, Wayne State University (Detroit, MI, USA), and the Detroit Medical Center (Detroit, MI, USA). Blood sample collection was performed from all women after obtaining written informed consent. The collection and use of biological specimens for research purposes was approved by the respective Institutional Review Boards of Wayne State University and the Detroit Medical Center (WSU IRB 031318MP2F). The present study included pregnant women (*n* = 20), predominantly African American, whose peripheral blood was collected in the third trimester at a median gestational age of 39.1 (ranging from 37.4 – 41) weeks prior to the onset of labor or administration of any medication. The control study group comprised healthy non-pregnant women (*n* = 20) of reproductive age from the same community, of whom all except one had never been pregnant.

### Stimulation of peripheral blood mononuclear cells with viral ligands

Peripheral blood samples were obtained by venipuncture and collected into EDTA tubes. Peripheral blood mononuclear cells (PBMCs) were isolated using the Lymphoprep density gradient medium (Cat# 07801; StemCell Technologies Inc., Vancouver, Canada), per the manufacturer’s instructions. Isolated PBMCs were cultivated in RPMI 1640 Medium (Cat# 11875–093; Thermo Fisher Scientific, Life Technologies Limited, Paisley, UK) supplemented with 5% human serum (Cat# H3667; Sigma-Aldrich, St Louis, MO, USA) and 1% Penicillin–Streptomycin (Cat# 15140122; Thermo Fisher Scientific). The cells were plated onto cell culture plates at a density of 1 × 10^6^ cells/mL prior to treatment. For viral ligand stimulation, PBMCs were individually incubated with 2.5 µg/mL R848 (TLR7/8-based adjuvant; Cat# vac-r848; InvivoGen, San Diego, CA, USA), 1 µM Gardiquimod (TLR7 ligand; Cat# tlrl-gdqs; InvivoGen), 10 µg/mL Poly(I:C) (HMW) VacciGrade™ (TLR3-based adjuvant; Cat# vac-pic; InvivoGen), 50 µg/mL Poly(I:C) (HMW) LyoVec™ (RIG-I/MDA-5 ligand; Cat# tlrl-piclv; InvivoGen), and 2 µg/mL ODN 2216 (TLR9 ligand; Cat# tlrl-2216; InvivoGen) at 37 °C with 5% CO_2_ for 24 h with the addition of protein transport inhibitor cocktail (Cat# 00-4980-03; ThermoFisher Scientific) for the last 4 h of incubation. Following incubation, the isolated PBMCs were gently collected using a cell scraper and centrifuged at 300 × g and 4 °C for 5 min. Finally, the resulting cell supernatants from PBMCs were stored at -80 °C prior to cytokine profiling, while the cell pellets were immediately processed for immunophenotyping.

### Immunophenotyping

Collected PBMC pellets were resuspended in 1X phosphate-buffered saline (PBS; Life Technologies Limited, Pailey, UK) and incubated with 1 µL/mL of Fixable Viability Stain 510 (Cat# 564406; BD Biosciences, Franklin Lakes, NJ, USA) in the dark at room temperature for 15 min. Next, cells were washed and resuspended in FACS Stain Buffer (Cat# 554656; BD Biosciences). Extracellular anti-human monoclonal antibodies (Supplementary Table 1) were added to the cell suspensions, which were incubated in the dark at 4 °C for 30 min. Cells were then fixed and permeabilized using the BD Cytofix/Cytoperm Kit (Cat# 554714; BD Biosciences), according to the manufacturer’s instructions. Following permeabilization, intracellular anti-human monoclonal antibodies (Supplementary Table 1) were added to cell suspensions, which were incubated in the dark at 4 °C for 30 min. Finally, the cells were washed and resuspended in 0.5 mL of FACS Stain Buffer and acquired using the BD LSR Fortessa flow cytometer (BD Biosciences) with FACSDiva 9.0 software (BD Biosciences). FlowJo software version 10 (TreeStar, Ashland, OR, USA) was used to perform data analysis and create figures. Monocytes were identified as CD14^+^ cells. As shown in Supplementary Fig. 1, monocyte subsets were classified as follows: classical monocytes (CD14^hi^CD16^−^), intermediate monocytes (CD14^hi^CD16^+^), non-classical monocytes (CD14^lo^CD16^+^), and CD14^lo^CD16^−^ monocytes. Additional markers (Supplementary Table 1) were used to further immunophenotype cells within the identified subsets.

### Interferon profile of viral ligand-stimulated PBMCs

PBMCs were isolated, cultured, and the resulting cell supernatants were collected as previously described. The concentrations of interferons were determined in cell supernatants using the U-PLEX Interferon Combo (human) (Cat# K15094K-1; Meso Scale Discovery, Rockville, MD, USA), following the manufacturer’s instructions. The following immune mediators were assayed: IFN-α2a, IFN-β, IFN-γ, and IL-29/IFN-λ1. A MESO QuickPlex SQ 120 was used to read the plates, and cytokine concentrations were calculated using the Discovery Workbench software version 4.0 (Meso Scale Discovery). The assay sensitivities were: 4 pg/mL (IFN-α2a), 3.1 pg/mL (IFN-β), 1.7 pg/mL (IFN-γ), and 1.2 pg/mL (IL-29/IFN-λ1).

### Statistical analyses

The R statistical programming language was used to perform all statistical analyses. Linear mixed effects models were fit for the comparison of flow cytometry data and cytokine concentrations between groups to account for repeated measurements. The data obtained by flow cytometry were modeled as frequencies. A false discovery rate adjusted *p*-value (q-value) < 0.05 was considered statistically significant. Differences in proportions of monocytes subsets are represented as heatmaps, and selected significant comparisons are displayed as box and whiskers plots. GraphPad Prism version 9.5.1 for Windows (GraphPad Software, San Diego, California, USA, www.graphpad.com) was used to conduct statistical analysis to evaluate differences in interferon concentrations using the Kruskal–Wallis test with post hoc multiple comparisons. A *p*-value < 0.05 was considered statistically significant.

## Results

### TLR8 drives the response to ssRNA stimulation in pregnancy-derived monocytes

**Fig. 1:**
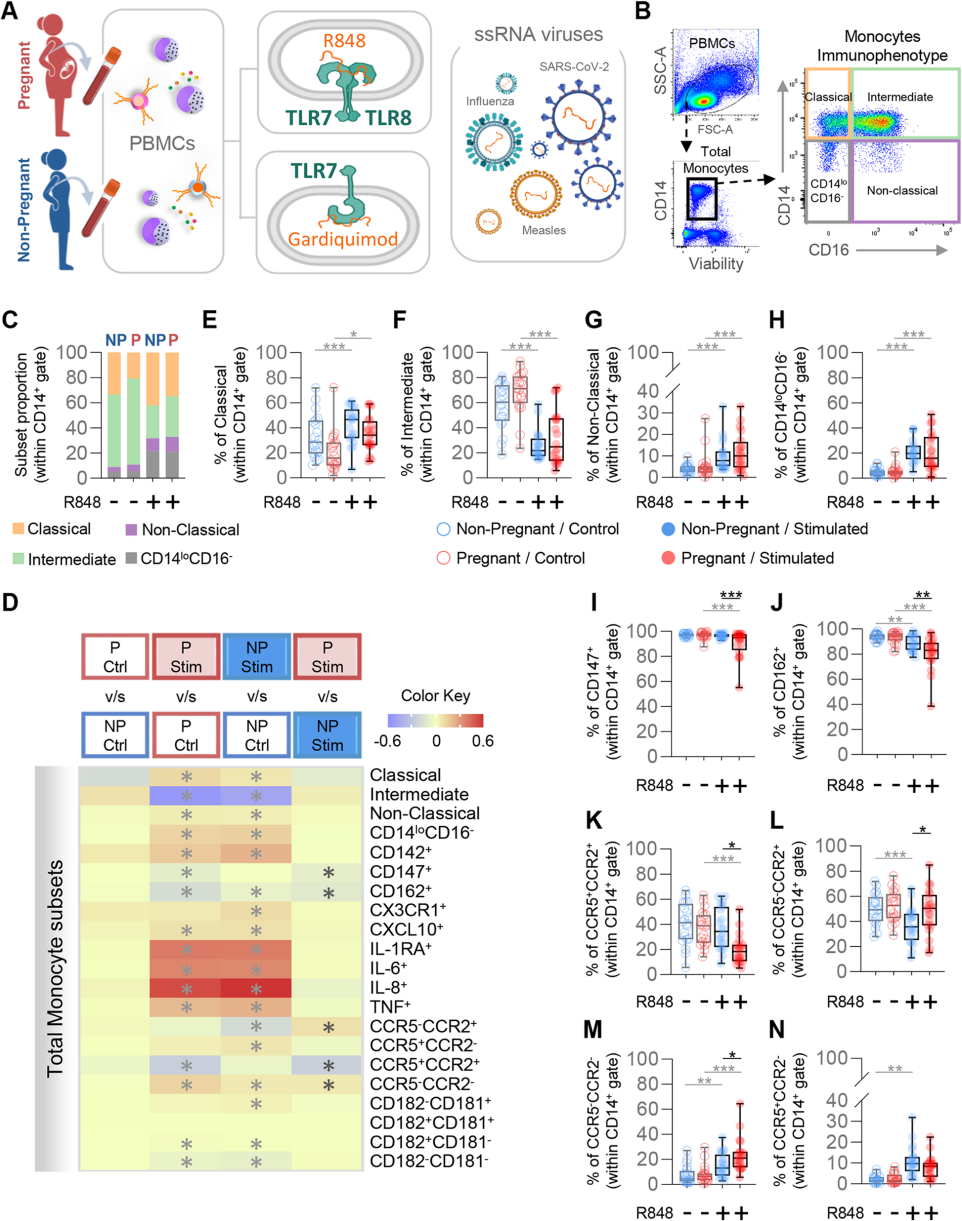
Monocyte response to TLR7/TLR8 stimulation. **A** Peripheral blood mononuclear cells (PBMCs) were isolated from the peripheral blood of pregnant (*n *= 20) and non-pregnant (*n* = 20) women and stimulated with R848 (TLR7/TLR8 agonist) or Gardiquimod (TLR7 agonist) for 24 h. Flow cytometry was performed to phenotype monocytes. **B** Flow cytometry gating strategy for phenotyping of monocyte subsets after in vitro stimulation with viral ligands. Viable monocytes were gated as live CD14^+^ cells from PBMCs. The expression levels of CD16 and CD14 were used to gate monocyte subsets as follows: classical (CD14^hi^CD16^−^); intermediate (CD14^hi^CD16^+^); non-classical (CD14^lo^CD16^+^), and CD14^lo^CD16^−^. **C** Proportions of monocyte subsets in pregnant (red) and non-pregnant (blue) women with and without R848 stimulation. **D** Heatmap representation of the differences in proportions of monocytes subsets from pregnant (red symbols) and non-pregnant (blue symbols) following R848 stimulation. Asterisks indicate statistically significant differences between the indicated groups. **E**-**N** Frequencies of (**E**) classical monocytes, (**F**) intermediate monocytes, (**G**) non-classical monocytes, (**H**) CD14^lo^CD16^−^ monocytes, (**I**) CD147^+^ monocytes, (**J**) CD162^+^ monocytes, (**K**) CCR5^+^CCR2^+^ monocytes, (**L**) CCR5^−^CCR2^+^ monocytes, (**M**) CCR5^−^CCR2^−^ monocytes, and (**N**) CCR5^+^CCR2^−^ monocytes in pregnant (red) and non-pregnant (blue) women following R848 stimulation (solid circles) or control (open circles). *p < 0.05; **p < 0.01; ***p < 0.001. ( +) Stimulated; (-) Control

Infection with single-stranded RNA (ssRNA) viruses such as rubella, enterovirus, measles, mumps, ebola, HIV, influenza, and coronaviruses has been linked to increased risk of adverse pregnancy outcomes [8, 61–65]. Since ssRNA genetic material can be sensed by TLR7 and TLR8, we first aimed to investigate whether stimulation with synthetic ligands for these receptors elicits a differential response during pregnancy. PBMCs were isolated from pregnant and non-pregnant women and stimulated with R848, an agonist of both TLR7 and TLR8 (Fig. 1A). Total monocytes (CD14^+^ cells) as well as classical (CD14^hi^CD16^−^), intermediate (CD14^hi^CD16^+^), non-classical (CD14^lo^CD16^+^), and CD14^lo^CD16^−^ monocytes were evaluated by flow cytometry (Fig. 1B). The proportions of each monocyte subset displayed similar shifts upon R848 stimulation for both pregnant and non-pregnant women (Fig. 1C). Differential responses after R848 stimulation were observed for pregnant- and non-pregnant-derived circulating monocytes, as shown in the heatmap representation in Fig. 1D. Specifically, exposure to R848 induced a significant change in the same direction and of similar magnitude in both pregnant- and non-pregnant-derived classical (Fig. 1E), intermediate (Fig. 1F), non-classical (Fig. 1G) and CD14^lo^CD16^−^ monocytes (Fig. 1H). By contrast, R848-stimulated monocytes from pregnant women showed reduced proportions of CD147^+^ (Fig. 1I), CD162^+^ (Fig. 1J), and CCR5^+^CCR2^+^ (Fig. 1K) cells as well as increased proportions of CCR5^−^CCR2^+^ (Fig. 1L) and CCR5^−^CCR2^−^ (Fig. 1M) cells compared with those from non-pregnant women, suggesting subtle pregnancy-driven differences in the monocyte response. No differences were found between pregnant- and non-pregnant-derived CCR5^+^CCR2^−^ monocytes (Fig. 1N) upon R848 stimulation. Taken together, these findings demonstrate that monocytes from pregnant and non-pregnant women respond to TLR7 and TLR8 stimulation. Yet, pregnancy is associated with reduced proportions of cells expressing adhesion molecules such as CD147 and CD162, as well as diminished proportions of cells expressing both CCR2 and CCR5 in response to TLR7/TLR8 stimulation.

**Fig. 2:**
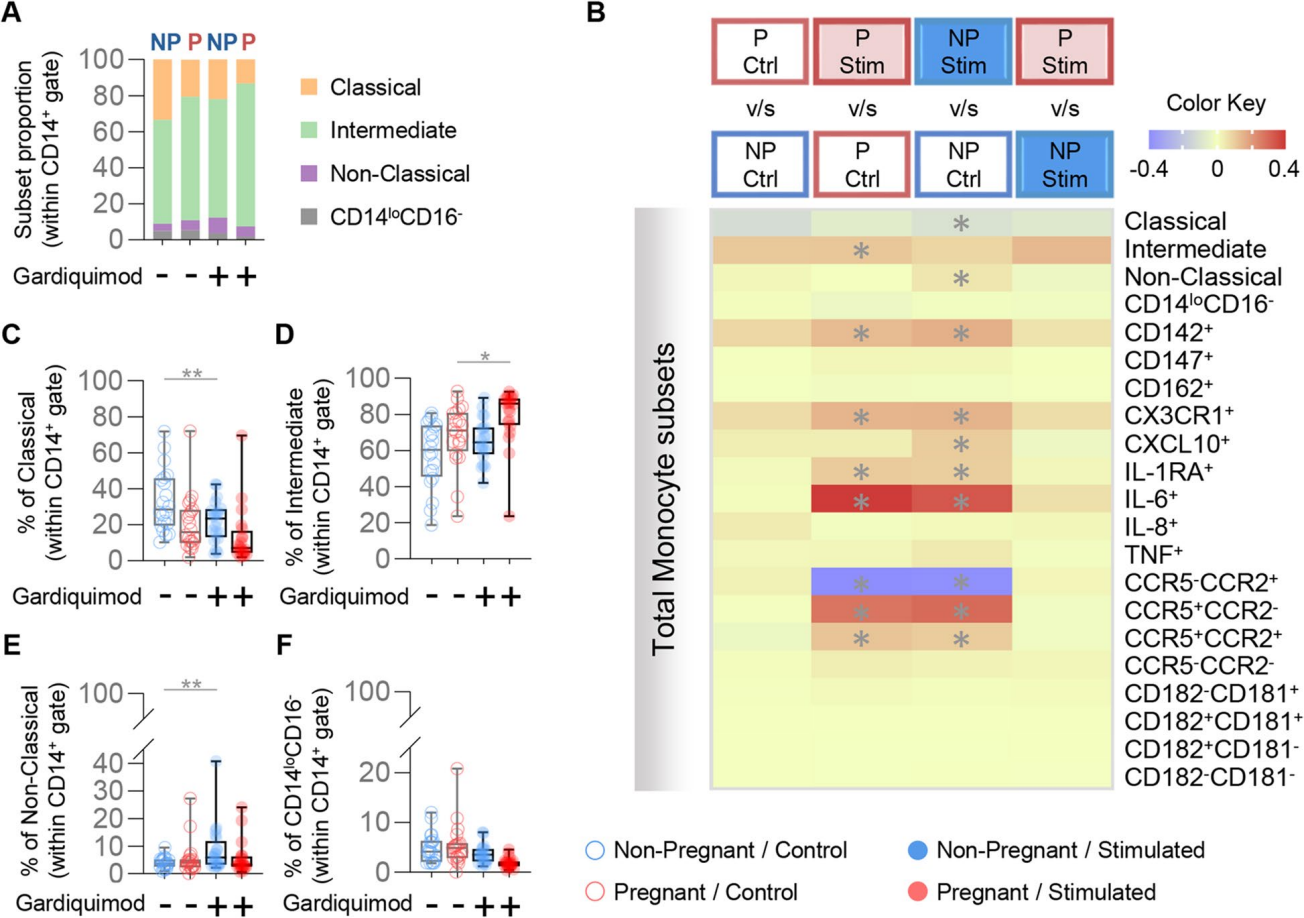
Monocyte response to TLR7 stimulation. **A** Proportions of monocyte subsets in pregnant (red) and non-pregnant women (blue) with and without Gardiquimod stimulation (TLR7 agonist). **B** Heatmap representation of the differences in proportions of monocyte subsets from pregnant (red symbols) and non-pregnant (blue symbols) women following Gardiquimod stimulation. Asterisks indicate statistically significant differences between the indicated groups. **C**-**F** Frequencies of (**C**) classical monocytes (CD14^hi^CD16^−^), (**D**) intermediate monocytes (CD14^hi^CD16^+^), (**E**) non-classical monocytes (CD14^lo^CD16^+^), (**F**) CD14^lo^CD16^−^ monocytes in pregnant (red) and non-pregnant (blue) women following Gardiquimod stimulation (solid circles) or control (open circles). *p < 0.05; **p < 0.01. ( +) Stimulated; (-) Control

We next used the specific TLR7 agonist Gardiquimod to distinguish responses specific to this PRR from those driven by either TLR7 or TLR8 (Fig. 1A). We found that TLR7-specific stimulation resulted in distinct effects on pregnant and non-pregnant-derived monocyte subsets (Fig. 2A). Overall, TLR7 stimulation induced more specific alterations of monocyte subpopulations (Fig. 2B) compared to the broad effects of R848 (Fig. 1D). Although the exposure to TLR7 stimulation differentially affected the proportions of pregnant and non-pregnant-derived classical (Fig. 2C), intermediate (Fig. 2D), and non-classical (Fig. 2E) monocytes, no pregnancy-specific differences were found between stimulated monocytes. Moreover, CD14^lo^CD16^−^ monocytes derived from both pregnant and non-pregnant women showed no change in proportion in response to TLR7 stimulation (Fig. 2F). Of note, the proportions of TLR7 stimulated monocytes expressing specific adhesion molecules or chemokine receptors did not differ between pregnant and non-pregnant women. Taken together, these results suggest that the differential responses observed in monocytes from pregnant women upon exposure to the TLR7/8 agonist are primarily driven by TLR8 stimulation.

### Pregnancy modulates the monocyte response to dsRNA

**Fig. 3:**
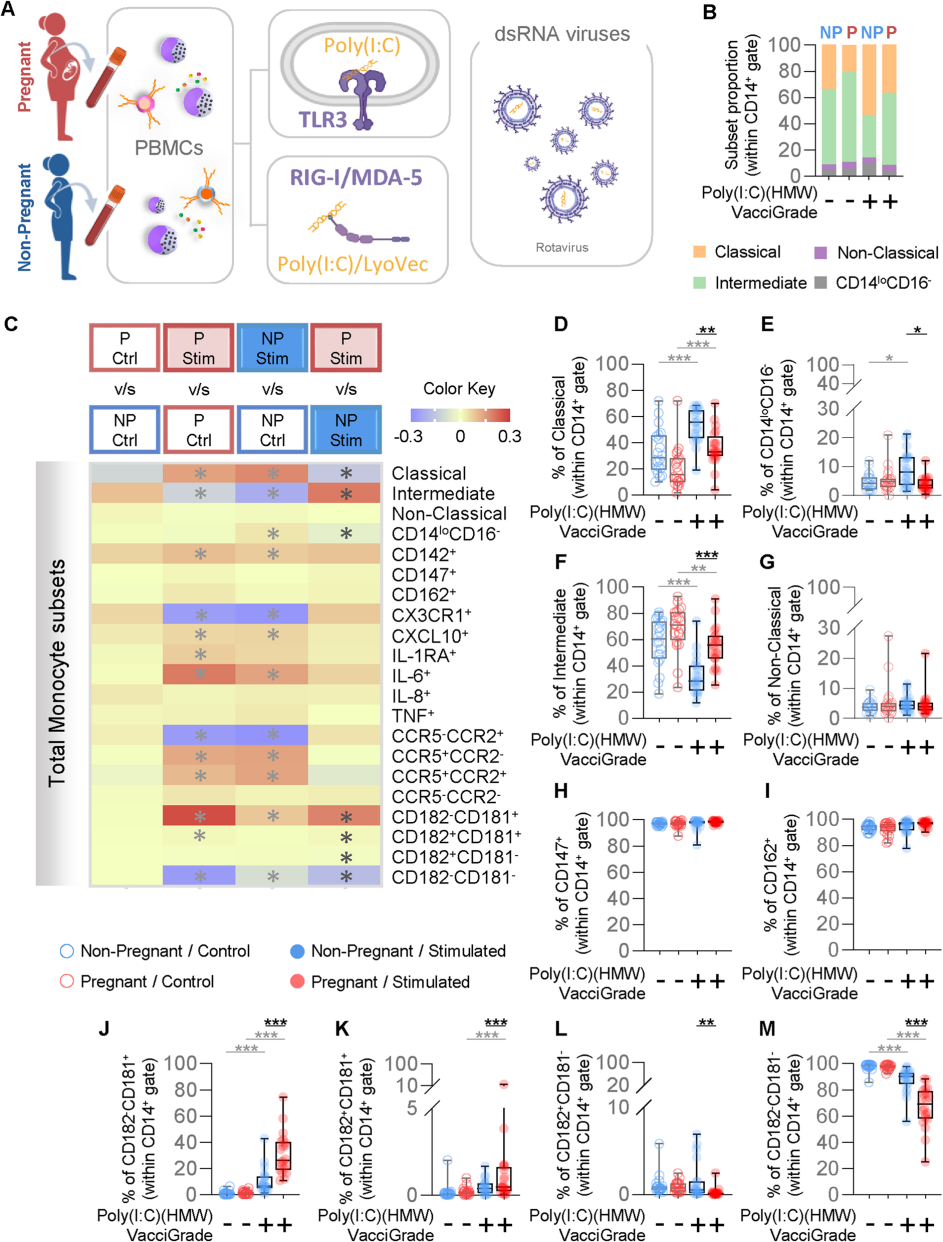
Monocyte response to TLR3 stimulation. **A** Peripheral blood mononuclear cells (PBMCs) were isolated from the peripheral blood of pregnant (*n* = 20) and non-pregnant (*n *= 20) women and stimulated with Poly(I:C) (HMW) Vaccigrade™ (poly(I:C); TLR3 agonist) or Poly(I:C) (HMW)LyoVec™ (poly(I:C)/LyoVec; RIG-I/MDA5 agonist) for 24 h. Flow cytometry was performed to phenotype monocytes. **B** Proportions of monocyte subsets in pregnant (red) and non-pregnant (blue) women with and without Poly(I:C) (HMW) Vaccigrade™ stimulation. **C** Heatmap representation of the differences in proportions of monocyte subsets from pregnant (red symbols) and non-pregnant (blue symbols) following Poly(I:C) (HMW) Vaccigrade™ stimulation. Asterisks indicate statistically significant differences between the indicated groups. (**D**-**M**) Frequencies of (**D**) classical monocytes (CD14^hi^CD16^−^), (**E**) CD14^lo^CD16^−^ monocytes, (**F**) intermediate monocytes (CD14^hi^CD16^+^), (**G**) non-classical monocytes (CD14^lo^CD16^+^), (**H**) CD147^+^ monocytes, (**I**) CD162^+^ monocytes, (**J**) CD182^−^CD181^+^ monocytes, (**K**) CD182^+^CD181^+^ monocytes, (**L**) CD182^+^CD181^−^ monocytes, and (**M**) CD182^−^CD181^−^ monocytes in pregnant (red) and non-pregnant (blue) women following Poly(I:C) stimulation (solid circles) or control (open circles). *p < 0.05; **p < 0.01; ***p < 0.001. ( +) Stimulated; (-) Control

Double-stranded RNA (dsRNA) viruses, such as rotavirus, are known to cause gastroenteritis in non-pregnant individuals [66, 67]. However, the maternal immune response to this viral infection and the subsequent transfer of protective antibodies to the offspring play a key role in the prevention of severe neonatal disease, particularly in premature neonates [68]. Therefore, we next evaluated the response of circulating monocytes to dsRNA-based viral ligands. dsRNA structures are sensed by the endosomal TLR3 or cytosolic RIG-I/MDA5 receptors, depending on the intracellular site of detection. Specific stimulation of endosomal or cytosolic receptors can be achieved separately using only dsRNA structures (such as poly(I:C)) or dsRNA structures combined with a transfecting reagent (Poly(I:C) (HMW) LyoVec™), respectively. Thus, PBMCs isolated from the peripheral blood of pregnant and non-pregnant women were stimulated with poly(I:C) (HMW) Vaccigrade™ (TLR3 agonist) or poly(I:C) (HMW) LyoVec™ (RIG-I/MDA5 agonist) (Fig. 3A). The proportions of primary monocyte subsets (Fig. 3B) were skewed upon TLR3 stimulation in both study groups, with classical and intermediate monocyte subsets being enhanced or diminished, respectively (Fig. 3C). Moreover, the proportions of CD142^+^, CXCL10^+^, IL-6^+^, CCR5^+^CCR2^−^, CCR5^+^CCR2^+^, and CD182^−^CD181^+^ monocytes were increased in both groups in response to stimulation, while the CX3CR1^+^, CCR5^−^CCR2^+^, and CD182^−^CD181^−^ monocyte subsets were reduced (Fig. 3C). Interestingly, we observed reduced proportions of classical (Fig. 3D) and CD14^lo^CD16^−^ monocytes (Fig. 3E), as well as an increased proportion of intermediate monocytes (Fig. 3F) in pregnant women compared to non-pregnant women upon TLR3 stimulation. No changes in the proportion of non-classical monocytes were observed (Fig. 3G). While the proportions of monocytes expressing CD147 (Fig. 3H) or CD162 (Fig. 3I) were not modified by TLR3 stimulation, pregnancy was associated with differential changes in the proportions of monocytes expressing the IL-8 receptors CD181 and CD182 [69, 70] as well as monocytes lacking these markers (Figs. 3J-M). Specifically, the proportion of pregnancy-derived monocytes expressing CD181 alone (Fig. 3J) or in combination with CD182 (Fig. 3K) was increased in response to TLR3 stimulation compared to cells isolated from non-pregnant women.

**Fig. 4:**
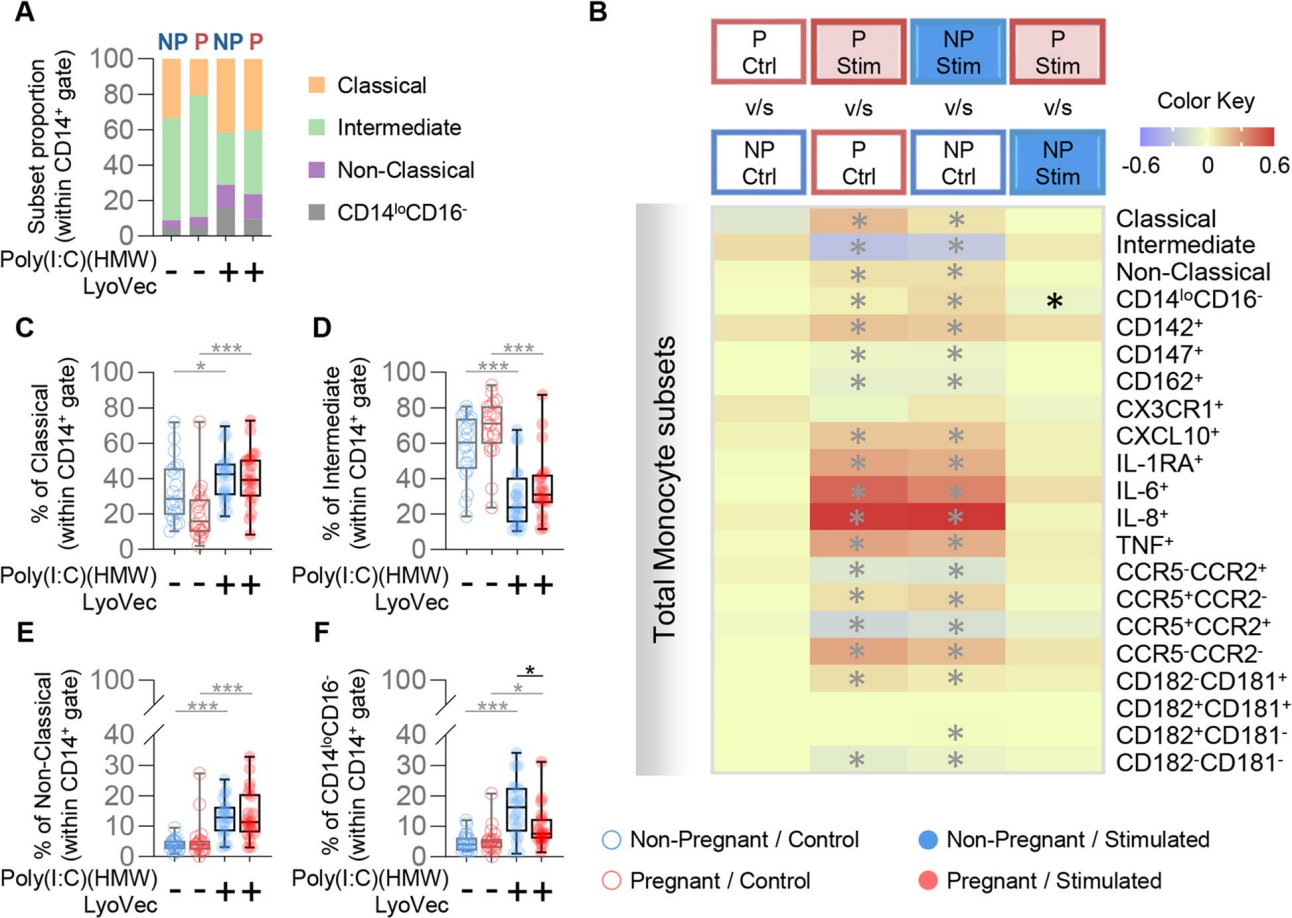
Monocyte response to RIG-I/MDA-5 stimulation. **A** Proportions of monocyte subsets in pregnant (red) and non-pregnant women (blue) with and without Poly(I:C) (HMW) LyoVec™ stimulation. **B** Heatmap representation of the differences in proportions of monocyte subsets from pregnant (red symbols) and non-pregnant (blue symbols) women following 24 h Poly(I:C) (HMW) LyoVec™ stimulation. Asterisks indicate statistically significant differences between the indicated groups. **C**-**F** Frequencies of (**C**) classical monocytes (CD14^hi^CD16^−^), (**D**) intermediate monocytes (CD14^hi^CD16^+^), (**E**) non-classical monocytes (CD14^lo^CD16^+^), and (**F**) CD14^lo^CD16^−^ monocytes in pregnant (red) and non-pregnant (blue) women following Poly(I:C) (HMW) LyoVec™ stimulation (solid circles) or control (open circles). *p < 0.05; ***p < 0.001. ( +) Stimulated; (-) Control

Similar to the results of TLR3 stimulation, the proportions of primary monocyte subsets showed elevated proportions of classical, non-classical, and CD14^lo^CD16^−^ monocytes together with reduced intermediate monocytes after exposure to RIG-I/MDA5 stimulation (Fig. 4A). Moreover, this cytosolic dsRNA agonist induced an extensive phenotypic response in monocytes from both pregnant and non-pregnant women (Fig. 4B); yet, pregnancy was not associated with differential effects for the majority of evaluated subsets. Indeed, the proportions of classical (Fig. 4C), intermediate (Fig. 4D), and non-classical (Fig. 4E) monocytes showed comparable responses between pregnant and non-pregnant samples stimulated with the RIG-I/MDA5 agonist. Only the proportion of CD14^lo^CD16^−^ monocytes subset differed between pregnant and non-pregnant women, with CD14^lo^CD16^−^ monocytes from non-pregnant women showing increased proportions in response to stimulation (Fig. 4F). Taken together, these findings suggest that pregnancy does not greatly modify the monocyte response to intracellular dsRNA; yet, the recognition of this dsRNA by endosomal TLR3 induces a distinct phenotype in monocytes from pregnant women.

### Pregnancy does not modify the monocyte response to dsDNA

**Fig. 5:**
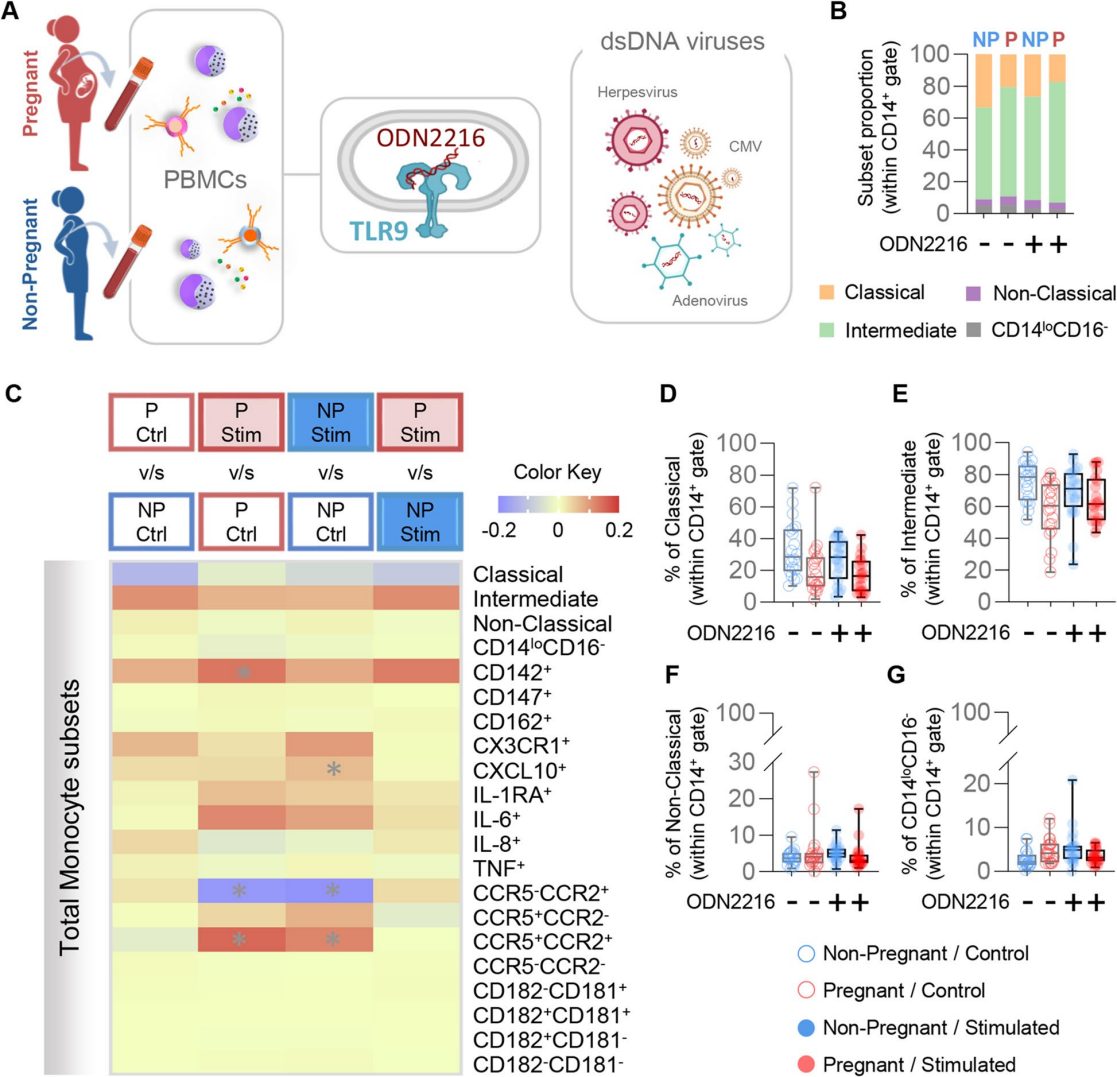
Monocyte response to TLR9 stimulation. **A** Peripheral blood mononuclear cells (PBMCs) were isolated from the peripheral blood of pregnant (*n* = 20) and non-pregnant (*n *= 20) women with and without ODN2216 stimulation for 24 h. Flow cytometry was performed to evaluate the total monocyte subsets. **B** Proportions of monocyte subsets in pregnant (red) and non-pregnant (blue) women with ODN2216 stimulation. **C** Heatmap representation of the differences in proportions of monocyte subsets from pregnant (red symbols) and non-pregnant (blue symbols) women following ODN2216 stimulation. Asterisks indicate statistically significant differences between the indicated groups. **D**-**G** Frequencies of (**D**) classical monocytes (CD14^hi^CD16^−^), (**E**) intermediate monocytes (CD14^hi^CD16^+^), **(F)** non-classical monocytes (CD14^lo^CD16^+^), and (**G**) CD14^lo^CD16^−^ monocytes in pregnant (red) and non-pregnant (blue) women following ODN2216 stimulation (solid circles) or control (open circles). ( +) Stimulated; (-) Control

Among double-stranded DNA (dsDNA) viruses, adenovirus, CMV, and herpesvirus infections have each been associated to increased risk for pregnancy complications such as fetal death or preterm birth, among others [3, 71, 72]. Therefore, we last evaluated the circulating monocyte response to dsDNA structures using the synthetic TLR9 agonist ODN2216 (Fig. 5A). Notably, the proportions of the primary monocyte subsets were largely unaltered upon stimulation with the TLR9 agonist, regardless of pregnancy status (Fig. 5B). Nonetheless, TLR9 stimulation enhanced the proportions of monocytes with a CCR5^+^CCR2^+^ phenotype while diminishing the CCR5^−^CCR2^+^ subset in both study groups (Fig. 5C). Other monocyte phenotypes also tended to be elevated in response to TLR9 stimulation, including CD142^+^, CX3CR1^+^, CXCL10^+^, IL-6^+^, and CCR5^+^CCR2^−^ cells; however, no pregnancy-specific differences were observed (Fig. 5C-G). These results are indicative of a consistent response towards dsDNA in circulating monocytes that is independent of pregnancy status.

### Pregnancy does not impair the release of interferons by mononuclear cells in response to RNA viral ligands

**Fig. 6:**
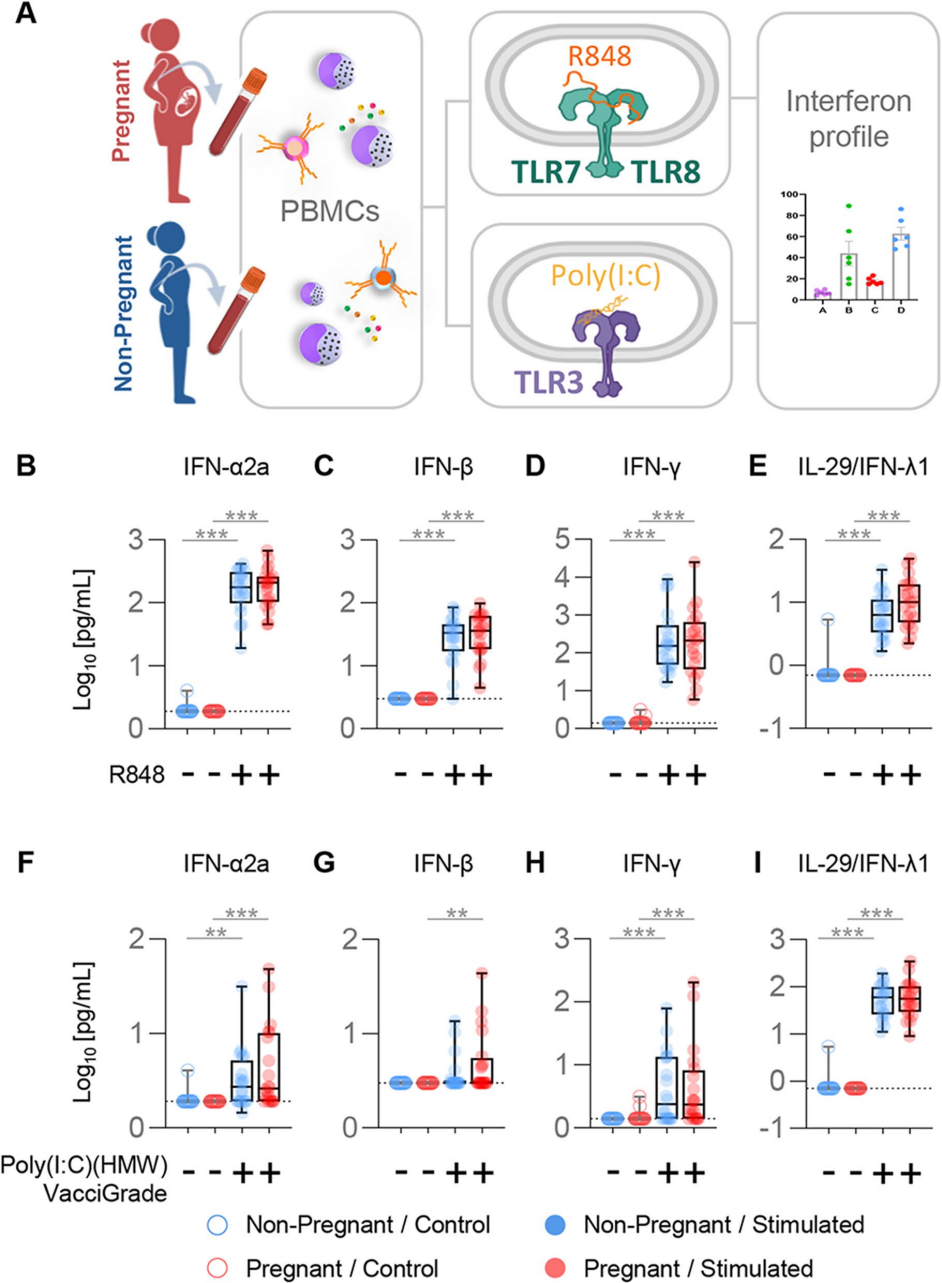
Interferon production by peripheral blood mononuclear cells (PBMCs) upon TLR7/TLR8 or TLR3 stimulation. **A** Peripheral blood samples were collected from pregnant (*n *= 20, indicated in red) and non-pregnant (*n* = 20, indicated in blue) women to isolate PBMCs for in vitro stimulation with R848 or Poly(I:C) (HMW) VacciGrade™ [Poly(I:C)]. Type-I (IFN-α2a, -β), Type-II (IFN-γ) and Type-III (IL-29/IFN-λ1) interferon concentrations were then determined in culture supernatants. **B**-**E** Log_10_-transformed concentrations of (**B**) IFN-α2a, (**C**) IFN-β, (**D**) IFN-γ, and (**E**) IL-29/IFN-λ1 in culture supernatants of PBMCs from pregnant (red symbols) and non-pregnant (blue symbols) women in response to R848 (solid circles) or control (open circles). **F**-**I** Log_10_-transformed concentrations of (**F**) IFN-α2a, (**G**) IFN-β, (**H**) IFN-γ, and (**I**) IL-29/IFN-λ1 in culture supernatants of PBMCs from pregnant (red symbols) and non-pregnant (blue symbols) women in response to Poly(I:C) (solid circles) or control (open circles). Dotted lines indicate the detection limit of each analyte. **p < 0.01, ***p < 0.001. ( +) Stimulated, (-) Control

Up to this point, our results indicate that pregnancy is associated with differential circulating monocyte responses upon exposure to RNA-based, but not DNA-based, viral ligands. Therefore, we last aimed to investigate whether the release of soluble immune mediators by mononuclear cells in response to RNA-based viral ligands differs between pregnant and non-pregnant women. As interferons (IFNs) are the primary early antiviral mediators secreted by immune and non-immune cells [73–76], we next measured the concentrations of IFN-α2a and IFN-β (type I IFNs), IFN-γ (type II IFN), and IL-29/IFN-λ1 (type III IFN) released upon in vitro stimulation of PBMCs with TLR7/8 or TLR3 agonists (Fig. 6A). The baseline production of IFNs was negligible by PBMCs from pregnant and non-pregnant women (Fig. 6B-I). Stimulation with a TLR7/TLR8 agonist (R848) resulted in elevated concentrations of IFN-α2a (Fig. 6B), IFN-β (Fig. 6C), IFN-γ (Fig. 6D), and IL-29/IFN-λ1 (Fig. 6E) released by PBMCs from pregnant and non-pregnant women. Consistently, concentrations of IFN-α2a (Fig. 6F), IFN-γ (Fig. 6H), and IL-29/IFN-λ1 (Fig. 6I) were increased in response to TLR3 stimulation (via poly(I:C) (HMW) Vaccigrade™). Notably, the latter stimulus resulted in elevated release of IFN-β by PBMCs from pregnant women, but not from non-pregnant women (Fig. 6G). Thus, our results suggest that circulating mononuclear cells from pregnant women retain the capacity to effectively release IFNs in response to viral stimulation.

## Discussion

Herein, we showed that the frequency of monocytes expressing the adhesion molecules CD147 and CD162 was diminished in pregnant women in response to TLR7/TLR8 stimulation. CD147, commonly termed Basigin, is a membrane receptor and member of the immunoglobulin superfamily that participates in cellular functions including migration and adhesion [77–80]. Similarly, CD162, or P-selectin glycoprotein ligand-1 (PSGL-1), acts as a ligand for selectins and is also a key player in leukocyte migration/adhesion [81–83]. Cellular adhesion molecules are among the primary points of cell entry for multiple viral families [84], and the modulation of such receptors can be a mechanism for viral pathogenicity. For example, the ssRNA Zika virus was shown to upregulate integrins and other adhesion molecules in monocytes, which potentially enhanced dissemination into neural cells [85]. Importantly, infection with ssRNA viruses during pregnancy is linked to increased risk of adverse outcomes and more severe clinical features compared to non-pregnant patients [8, 61, 63–65, 86–88]. Indeed, while enteroviruses are largely asymptomatic in non-pregnant patients, they have been shown to induce obstetric complications [64]. The enhanced downregulation of adhesion receptors upon TLR7/TLR8 stimulation in pregnancy-derived monocytes may thus represent a defensive strategy to slow viral entry and potentially protect the fetus at the expense of the mother.

The above concept is further supported by the distinct regulation of CCR5 and CCR2 expression in response to TLR7/TLR8 stimulation of pregnancy-derived monocytes observed herein. The chemokine receptors CCR5 and CCR2 are integral for mediating monocyte trafficking and inflammatory responses [89, 90], and thus the distinct changes in the distribution of pregnancy-derived monocytes expressing these receptors could help to explain the differing susceptibility to viral infection. Specifically, we found that monocytes with a double-positive CCR5^+^CCR2^+^ phenotype were diminished in pregnant women compared to non-pregnant, while CCR5^−^CCR2^+^ and CCR5^−^CCR2^−^ subsets were more abundant, suggesting a tendency for enhanced downregulation of these chemokine receptors during viral stimulation. Importantly, CCR5 has been implicated as a co-receptor in viral cell entry by the ssRNA virus HIV-1 [91–95], with the expression levels of this receptor being directly associated with the rates of monocyte/macrophage infection [96]. Together, these findings provide evidence for distinct modulation of monocyte phenotypes in response to ssRNA viral stimulation during pregnancy that may serve to protect the fetus from vertical transmission. However, the reduced abundance of cells expressing adhesion molecules and chemokine receptors in monocytes from pregnant women exposed to ssRNA viruses may also disrupt the capacity of these immune cells to migrate to sites of infection/inflammation.

Notably, we also found that stimulation of monocytes via TLR7 alone was not associated with differences between pregnant and non-pregnant individuals, suggesting that the differential effects of ssRNA viruses in monocytes from pregnant women are mediated primarily through TLR8. This concept is supported by a previous in vitro investigation of the relationship between placental growth factor-1 (PlGF-1), which increases in the maternal circulation during pregnancy and peaks in the third trimester [97], and CD14^+^ cellular responses to TLRs stimulation [98]. It was observed that TNF release was enhanced when TLR8 stimulation occurred in the presence of PlGF-1. Moreover, while targeted TLR7 stimulation in the presence of PIGF-1 triggered a mild increase in TNF production, the combined stimulation of TLR7/TLR8 induced the strongest effect [98]. These results suggest that pregnancy-specific physiologic changes can modulate TLR signaling pathways in monocytes, including a greater responsiveness to ssRNA-mediated TLR8 stimulation, which may contribute to an enhanced maternal response to viral infections such as HIV, influenza, and coronaviruses. It is worth mentioning that, despite their shared recognition of ssRNA viral ligands, TLR7 and TLR8 have been reported as modulating distinct signaling pathways in monocytes, resulting in the biased release of cytokines and interferons [99]. Thus, the observed greater dependence on TLR8 signaling for pregnancy-specific responses to ssRNA may have additional implications for subsequent mediator release by maternal monocytes that were not revealed by the analysis performed herein. Moreover, the potential combined action of TLR7 and TLR8 in monocytes has not been adequately investigated and thus the stimulation of both receptors, or crosstalk between their signaling pathways, may have additional effects that are not yet understood [99].

In the current study, we utilized poly(I:C) to induce TLR3 stimulation and thereby model dsRNA viral infection, and found that monocytes expressing CD181 (CD181^+^CD182^+^ and CD181^+^CD182^−^) were increased, while those without CD181 expression (CD181^−^CD182^+^ and CD181^−^CD182^−^) were diminished, in pregnant women compared to non-pregnant individuals. More commonly known as CXCR1 and CXCR2, these molecules act as receptors for multiple chemokines including CXCL1 and IL-8 [100–102]. A prior report indicated that poly(I:C) treatment in pregnant rats resulted in elevated concentrations of multiple mediators in the circulation, including monocyte chemoattractants such as CXCL1, CCL3, and CCL20 [103], supporting the participation of monocyte chemokine signaling pathways as part of the response to TLR3 stimulation. CXCR1 and CXCR2 have overlap in their recognized chemokines and were thought to induce similar functions that centered on neutrophil recruitment [89, 90]. However, reports have suggested that the downstream effects mediated by these receptors may differ; for example, while both CXCR1 and CXCR2 respond to IL-8 (CXCL8), the latter receptor undergoes rapid internalization compared to the former [104]. Thus, the biased modulation of these two chemokine receptors favoring CXCR1-expressing monocytes in response to TLR3 stimulation may indicate a pregnancy-specific program of immune regulation; yet, this concept requires further investigation.

TLR9 is an important component of host defense against dsDNA viruses [44–46]. Here, we report that stimulation of TLR9 resulted in a modest but consistent increase in intermediate monocytes as well as those expressing chemokine receptors and inflammatory cytokines/chemokines in both pregnant and non-pregnant samples. Our results are consistent with studies demonstrating that TLR9 mRNA is expressed in the murine uterus, cervix, and placenta throughout gestation [105], and that the protein expression of TLR9 in human peripheral leukocytes is unaltered by pregnancy [106]. Given the frequency of encountering dsDNA viruses such as CMV during pregnancy [107–111], the conservation of TLR9 signaling and its downstream effects may be important for ensuring a sufficient maternal immune response against such common viral threats. Interestingly, alterations in TLR9 signaling resulting from single nucleotide polymorphisms or the activation of this receptor via mtDNA have been linked to obstetrical pathologies such as preeclampsia and spontaneous preterm birth [112–114]. Moreover, TLR9 has also been proposed to respond to cell-free fetal DNA (cffDNA), small DNA fragments derived from placental cells and released into maternal circulation [115, 116], in the context of obstetrical disease [117–122], as demonstrated using animal models [123, 124], and in normal term parturition [125, 126]. In light of the reported link between TLR9 signaling and adverse pregnancy outcomes, it is likely that the downstream inflammatory cascade is tightly regulated under steady-state conditions. Regardless, further investigation is required to mechanistically investigate the role of TLR9 responses to pathogen-derived CpGs or cffDNA in pregnancy complications.

Interferons, which represent one of the first lines of soluble defense against pathogens and are critical for an effective anti-viral response, are divided into three types that differ according to their receptor complexes and signal transduction pathways [127]. Here, we determined the release of select Type I, II, and III interferons in response to TLR3 and TLR7/TLR8 stimulation, and noted substantial increases across all mediators in PBMCs from both pregnant and non-pregnant women. The maintenance of interferon signaling is particularly important during pregnancy to protect the fetus against potential congenital infection [128]. Yet, it is worth mentioning that a substantial proportion of human genes can potentially be differentially regulated by interferons; indeed, the family of interferon-stimulated genes (ISGs) continues to grow as new members are identified [129]. Thus, it is possible that, although the released IFN profile appears unchanged during pregnancy, the monocyte signature of ISGs that is modulated by viral signaling may undergo distinct regulation compared to non-pregnant individuals, resulting in a tailored immune response.

## Conclusions

Collectively, the data presented herein provide evidence that monocytes from pregnant women display a response to in vitro stimulation with viral ligands that is distinct from the non-pregnant state. Specifically, we demonstrate a differential expression pattern of adhesion molecules and chemokine receptors in pregnancy-derived monocytes exposed to ssRNA and dsRNA viral mimetics that is primarily driven by TLR8 and membrane-bound TLR3, respectively. By contrast, while stimulation of cytosolic TLR3 and TLR9 induced substantial changes in monocyte phenotypes, pregnancy-specific responses were not observed. Notably, the monocyte soluble interferon response to ssRNA or dsRNA viral stimulation remained intact in pregnant women. Taken together, our data provide insight into the specific modifications to the systemic immune response during pregnancy that may help explain the reduced capacity of pregnant women to counteract viral infection, as observed during recent and historic pandemics. The identification of signaling pathways implicated in the pregnancy-specific changes in monocyte phenotypes may allow for a more nuanced assessment of individual risk for infected pregnant women according to the type of virus. Moreover, our findings can serve as a foundation for future research to determine whether monocytes or their affected pathways can serve as targets for preventing or treating viral infection during pregnancy.

## Supplementary Information

**Additional file 1. Table S1.**

**Additional file 2. Figure S1.**

## Data Availability

All of the data generated or analyzed during this study are included in this published article or its supporting information.

## Abbreviations

CCL: C–C motif chemokine ligand
CCR: C–C motif chemokine receptor
cffDNA: Cell-free fetal DNA
CXCL: C-X-C motif chemokine ligand
CXCR: C-X-C motif chemokine receptor
dsRNA: Double-stranded RNA
HMW: High molecular weight
IFN: Interferon
MDA5: Melanoma differentiation-associated protein 5
mtDNA: Mitochondrial DNA
PBMC: Peripheral blood mononuclear cell
PSGL-1: P-selectin glycoprotein ligand-1
RIG-I: Retinoic acid-inducible gene I
ssRNA: Single-stranded RNA
TLR: Toll-like receptor
