# Supplementary material for: Differential immunophenotype of circulating monocytes from pregnant women in response to viral ligands
Source: BMC Pregnancy Childbirth. 2023 May 6;23:323. doi: 10.1186/s12884-023-05562-0 (PMC10163583; doi:10.1186/s12884-023-05562-0)
Supplement: Supplementary file 1 — Additional file 1. Table S1. [file 12884_2023_5562_MOESM1_ESM.docx]

**Supplementary Table 1.** List of anti-human antibodies used throughout the study.

| **Cell Marker** | **Fluorochrome** | **Isotype** | **Clone** | **Company** | **Cat #** |
| --- | --- | --- | --- | --- | --- |
| CD14 | BUV395 | Mouse BALB/c IgG2b, κ | MφP9 | BD Biosciences | 563562 |
| CD16 | APC-H7 | Mouse BALB/c x DBA/2 (also known as CD2F1 or CDF1) IgG1, κ | 3G8 | BD Biosciences | 560195 |
| CCR5 (CD195) | BV711 | Mouse C57BL/6 IgG2a, κ | 2D7/CCR5 | BD Biosciences | 563395 |
| CXCR1 (CD181) | PE-Cy5 | Mouse IgG2b, κ | 5A12 | BD Biosciences | 551081 |
| CXCR2 (CD182) | APC | Mouse IgG1, λ | 6C6 | BD Biosciences | 551127 |
| CD142 (Tissue Factor) | BUV737 | Mouse IgG1, κ | HTF-1 | BD Biosciences | 748835 |
| CD162 (PSGL) | BV786 | Mouse BALB/c IgG1, κ | KPL-1 | BD Biosciences | 743483 |
| CCR2 | BV650 | Mouse BALB/c IgG2a, κ | LS132.1D9 | BD Biosciences | 747849 |
| CD147 (Basigin) | PerCP-Cy5.5 | Mouse IgG1, κ | HIM6 | BD Biosciences | 562554 |
| CX3CR1 | PE-Cy7 | Rat IgG2b, κ | 2A9-1 | BioLegend | 341612 |
| IL-6 | PE-CF594 | Rat IgG1 | MQ2-13A5 | BD Biosciences | 563543 |
| IL-8 | BV421 | Mouse IgG2b | G265-8 | BD Biosciences | 563310 |
| TNF | BV605 | Mouse IgG1, κ | MAb11 | BioLegend | 502936 |
| CXCL10 | Alexa Fluor 488 | Mouse IgG1 | 33036 | R&D Systems | IC266G-100UG |
| IL-1RA | PE | Mouse IgG1 | AS17 | BD Biosciences | 340525 |
