# Supplementary material for: Differential immunophenotype of circulating monocytes from pregnant women in response to viral ligands
Source: BMC Pregnancy Childbirth. 2023 May 6;23:323. doi: 10.1186/s12884-023-05562-0 (PMC10163583; doi:10.1186/s12884-023-05562-0)
Supplement: Supplementary file 2 — Additional file 2. Figure S1. [file 12884_2023_5562_MOESM2_ESM.pdf]

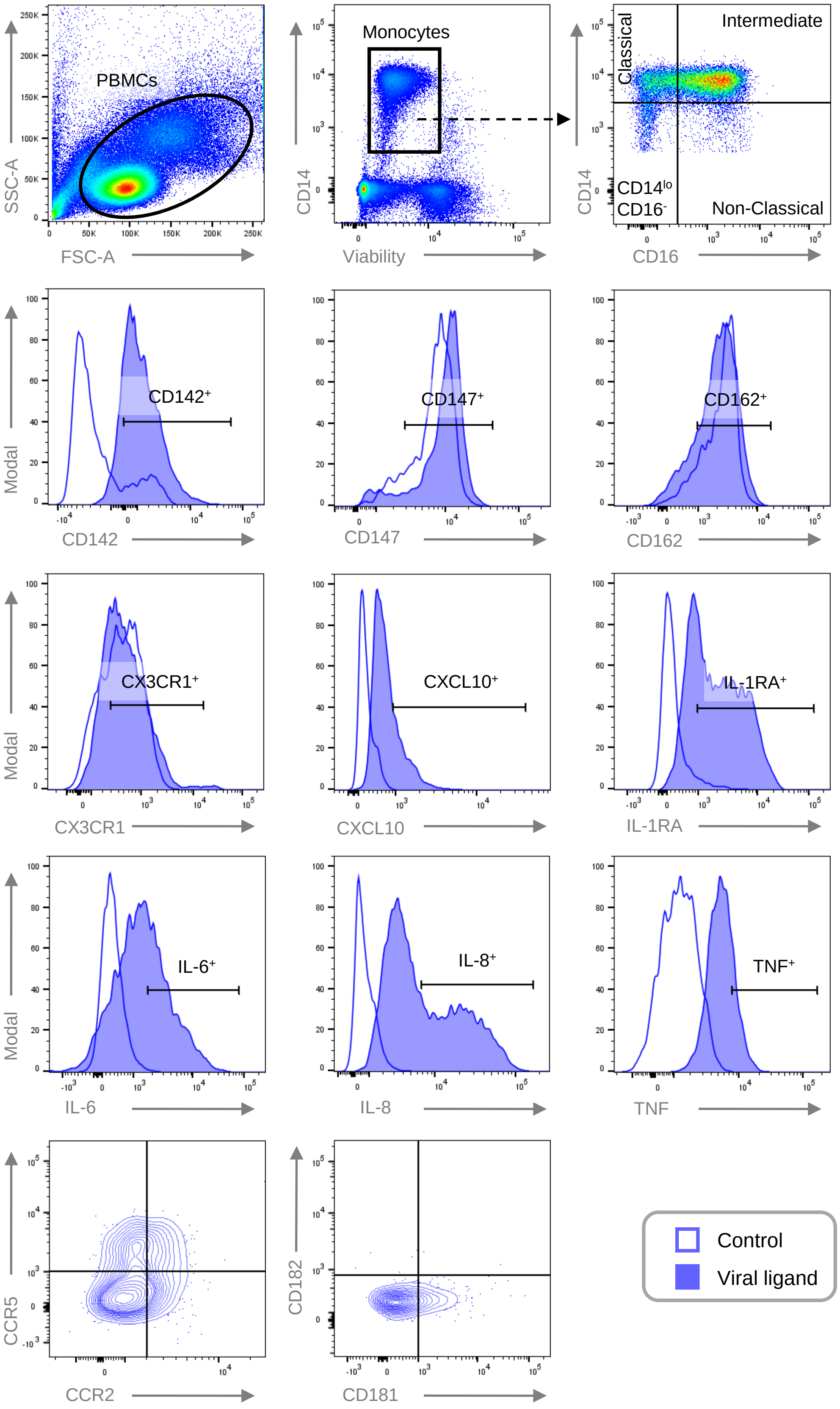

Supplementary Figure 1. Flow cytometry gating strategy for phenotyping of monocyte subsets upon viral ligand stimulation. Peripheral blood mononuclear cells (PBMCs) isolated from pregnant (n = 20) and non-pregnant (n = 20) women were gated for monocytes ($CD14^+$ cells, described in Fig. 1). The expression levels of CD16 and CD14 were used to gate monocyte subsets as follows: classical ($CD14^{hi}CD16^-$); intermediate ($CD14^{hi}CD16^+$); non-classical ($CD14^{lo}CD16^+$); and $CD14^{lo}CD16^-$. All four subsets as well as the total monocyte population were gated for specific phenotypes after 24 h of viral ligand stimulation (blue filled histograms) or control (blue open histograms).
